# From bedside to bytes: the digital transformation of the healthcare workforce

**DOI:** 10.3389/fdgth.2026.1777607

**Published:** 2026-03-19

**Authors:** Yiannis Kyratsis

**Affiliations:** Erasmus School of Health Policy and Management, Erasmus University Rotterdam, Rotterdam, Netherlands

**Keywords:** algorithmic governance, digital transformation, healthcare workforce, information-mediated work, organizational paradox, professional expertise, work design, work governance

## Abstract

Digital transformation is reshaping healthcare work, whereas research on workforce implications remains fragmented across disciplines. Effects like burnout, resistance, and workflow disruption are often framed as implementation failures rather than systematic outcomes of how work is reorganized. This Mini Review advances a four-dimensional analytical lens distinguishing work execution (task distribution, sequencing, temporal organization), work experience (autonomy, cognitive load, dignity), work governance (standardization, monitoring, accountability), and work learning and adaptation (workarounds, skill development, technology reshaping). The framework specifies information-mediated work, including documenting, coding, classifying, and verifying data, as the coupling mechanism binding these dimensions. This coupling is constitutive, in that documentation defines legitimate work;, transductive, in that changes spread across dimensions; and asymmetric, in that non-datafied work becomes invisible. Four characteristic paradoxes emerge: efficiency-intensification (execution–experience), empowerment–surveillance (experience–governance), innovation–compliance (learning–governance), and adaptation–deviation (learning–execution). These are structural features rather than design flaws, since informational practices that generate benefits in one dimension produce costs in another. Digital transformation also redistributes burdens unequally, concentrating execution demands, surveillance intensity, and learning constraints among lower-status workers. The framework reframes persistent tensions as predictable outcomes of dimensional misalignment rather than individual or technological shortcomings, and offers a diagnostic orientation for research and practice. Sustainable transformation depends on managing cross-dimensional trade-offs rather than eliminating them, with deliberate attention to whether digital systems support dignified, expert work.

## 1 Introduction

Digital transformation has become central to addressing efficiency, quality, and sustainability challenges in healthcare (1, 2). Technologies such as electronic health records (EHRs), clinical decision support systems, and artificial intelligence (AI) are promoted as solutions to rising demand, workforce shortages, and cost pressures (3–6). A substantial body of research documents the technical capabilities of these systems. However, their implications for the healthcare workforce remain fragmented across disciplines and are frequently treated as secondary implementation concerns.

Much existing literature approaches workforce outcomes through lenses of adoption, acceptance, or usability, implicitly privileging individual attitudes over the reorganization of work itself (7, 8). This narrow focus is echoed in broader debates calling for attention to organizational, social, and ethical dimensions of digital health (9). Healthcare quality research has documented unanticipated consequences including workflow disruption, burnout, alert fatigue, and new error pathways (10–12). These effects are often framed as implementation failures rather than systematic features arising from how work itself is reorganized (13).

Recent integrative reviews have advanced understanding of AI and work across sectors, highlighting effects on autonomy, skills, and wellbeing (14, 15). These contributions are largely sector-agnostic, while healthcare-specific studies offer rich empirical detail but remain weakly integrated at a theoretical level. This Mini Review responds by advancing a work-centered perspective on digital workforce transformation, identifying persistent blind spots in existing research, and outlining directions for a more robust research agenda.

## 2 Review approach

This Mini Review adopts an integrative, theory-building approach following conventions for eclectic reviews in management scholarship (16). The aim is purposive assembly of theoretically insightful sources rather than exhaustive coverage. Literature was identified through iterative searches across PubMed, Web of Science, and Scopus (2010–2025), supplemented by citation tracing. Inclusion criteria emphasized: empirical or conceptual focus on workforce outcomes; theoretical depth; and methodological rigor.

The synthesis comprises three categories. First, foundational contributions theorizing technology-work relationships, including sociomateriality (17), affordance theory (18), and algorithmic management (19). Second, empirical studies documenting work-level consequences in healthcare with analytic depth (20–22). Third, recent integrative reviews synthesizing cross-sectoral evidence (14). The review draws across technology types, settings, and professional groups to demonstrate applicability, though illustrative rather than comprehensive coverage was pursued given format constraints.

## 3 A four-dimensional analytical lens

**Figure 1:**
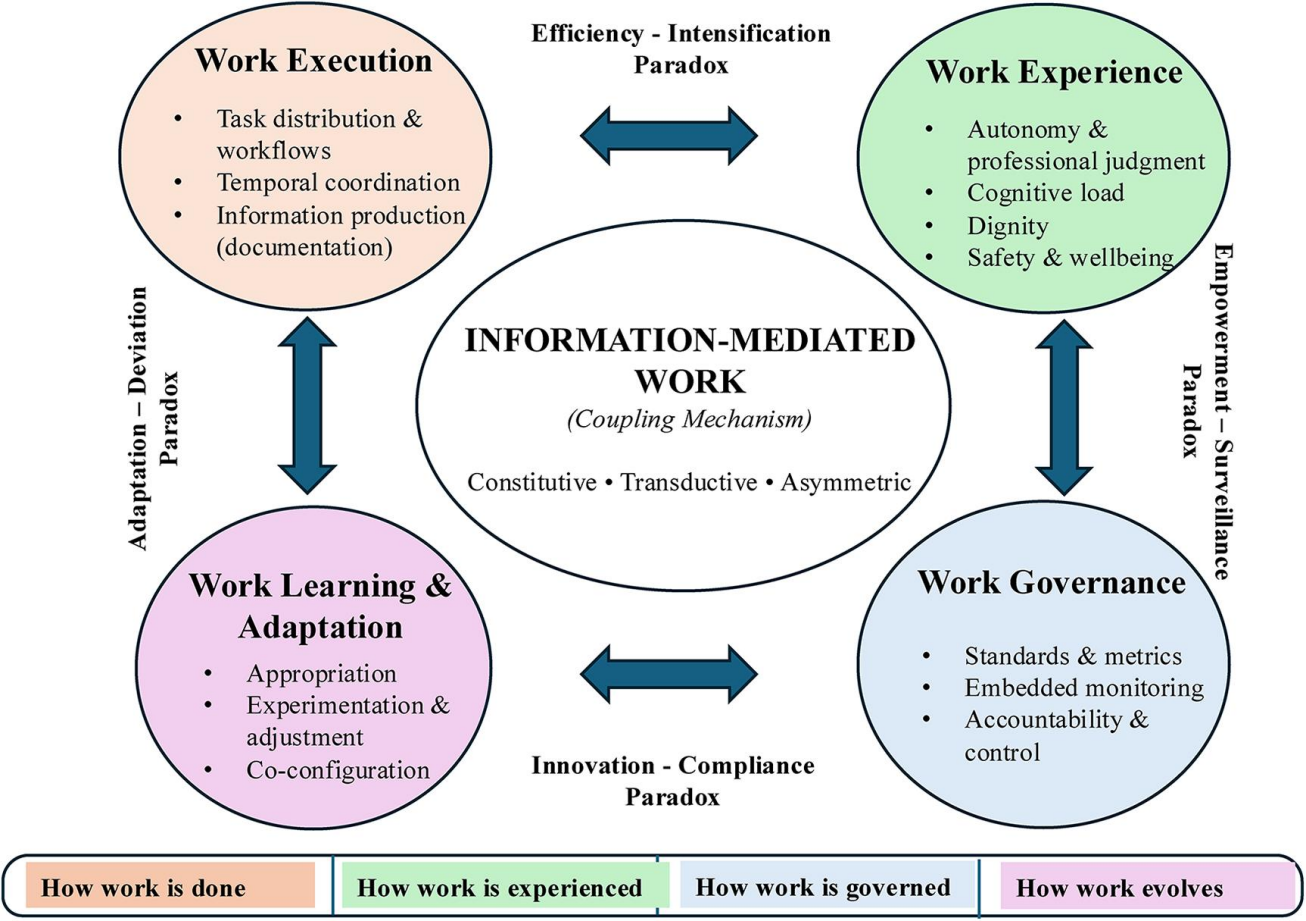
A four-dimensional analytical lens on digital workforce transformation.

Digital workforce transformation is conceptualized through a four-dimensional lens (Figure 1) distinguishing *work execution*, *work experience*, *work governance*, and *work learning and adaptation*. The contribution lies not in new labels but in analytically separating dimensions that are often conflated in research and in specifying how digital technologies bind them together in practice (17, 23).

Existing literature alternates between task-focused accounts [e.g., (18, 24)] experiential accounts of professional meaning [e.g., (22, 25)], and governance-centered analyses [e.g., (26)]. These remain siloed, obscuring how digital technologies simultaneously reorganize what work is done, how it is experienced, how it is governed, and how it evolves.

A central premise is that digital workforce transformation is organized through information-mediated work, including documenting, coding, classifying, verifying, and interpreting data. Information-mediated work functions as the coupling mechanism binding the four dimensions. This coupling operates through three properties: it is *constitutive,* in that documentation defines what counts as legitimate work, while undocumented care is organizationally invisible. It is *transductive,* in that changes propagates across dimensions through informational practices. It is *asymmetric,* in that work resisting datafication becomes systematically less visible.

Each dimension is associated with characteristic paradoxes that reflect structural features rather than design flaws. Table 1 maps illustrative studies onto these four dimensions, organized by technology cluster. This mapping shows coverage across technology types and professional groups while also revealing a consistent pattern in which existing research tends to concentrate within single dimensions rather than examining how changes spread across them. To support consistent application, each dimension can be operationally distinguished. Work execution concerns observable task content, task distribution, and temporal organization, describing what is done, by whom, and when. Work experience captures workers' cognitive, emotional, and moral responses, describing how work is perceived and lived. Work governance refers to mechanisms of standardization, monitoring, and accountability embedded in digital infrastructure. Work learning and adaptation encompasses ongoing processes of adjustment and reshaping, describing how digital work evolves through use. Although the same phenomenon may appear across dimensions, each directs attention to analytically distinct properties.

**Table 1:** Illustrative mapping of key studies to framework dimensions.

Cluster	Key studies	Technology type	Typical setting(s)	Main professions/actors	Primary dimensions informed
EHR - workload, workflow, burden	(10, 21, 27)	EHR	Primary care, ambulatory care	Physicians	Execution (time allocation, documentation, “desktop medicine”); Experience (workload, after-hours work)
EHR - implementation, disruption, workarounds	(3, 20, 28–30)	EHR/EPR	Hospitals, multi-setting	Physicians, nurses, multiple professionals	Execution (transition disruption, workflow reorganization, workarounds, boundary expansion); Governance (visibility, accountability); Learning (adaptation, workaround practices)
EHR/Health IT - safety & socio-technical frameworks	(12, 31–34)	Health IT, EHR, work systems	Multiple care settings	Multiple health professionals, managers, patients	Governance (safety, accountability, reporting); Execution (work system design, workflow alignment); Learning (human-centered redesign)
CDSS, alerts, ML-enabled devices, e-consults	(1, 11, 35, 36)	CDSS, ML devices, e-prescribing, e-consults	Inpatient care, outpatient care, integrated systems	Physicians, pharmacists, other clinicians	Execution (decision processes, alert load, digital prescribing workflows); Experience (alert fatigue); Governance (safety, oversight, accountability)
Telehealth, telemonitoring, self-care	(9, 13, 22, 36–38)	Telemonitoring, telehealth, self-monitoring, digital health	Community, heart-failure care, primary/secondary care, system-level programs	Patients, nurses, physicians, multiple professions	Experience (autonomy, proximity, recognition, ambivalence); Governance (conduct at a distance, visibility, adoption/scale-up); Execution (redistribution of tasks, new service models); Learning (embedding and spread)
Digital health bundles in clinical work	(6, 39)	Bundles of digital health tools (EHR, telehealth, analytics)	Hospitals, healthcare organizations	Physicians, nurses, managers, other staff	Execution (performance, workload, managerial support processes); Learning (skills and training needs); Governance (digital transformation strategies)
AI in clinical care and critical care	(2, 4, 35, 40)	AI/ML in diagnostics and critical care	Hospitals, ICUs, cross-specialty clinical settings	Clinicians, intensive care staff, organizations	Governance (regulation, safety, responsibility); Execution (decision augmentation, mitigating shortages, task reconfiguration); Learning (organizational capability to use AI)
AI opacity, explainability, and trust	(41, 42)	Diagnostic AI, explainable AI	Diagnostic work, multi-sector	Professionals and workers using AI tools	Experience (trust, dealing with opacity, relational impacts); Learning (collaboration with AI, sense-making); Governance (accountability for AI-assisted decisions)
Algorithmic management, metrics, “smart” systems	(15, 19, 24, 26, 43–45)	Algorithms, metrics/analytics, algorithmic management, smart systems	Cross-sector (incl. health-adjacent knowledge work)	Workers, professionals, knowledge workers	Governance (algorithmic control, surveillance, inequality, contested authority); Experience (dignity, dehumanization, meaning-making, resistance); Learning (adaptation to metrics and algorithms)
Professional work, identity, and digitalization	(25, 46, 47)	Digital and managerial technologies shaping professions	Healthcare and other professional fields	Professionals, especially clinicians	Governance (professional autonomy vs. managerial control); Experience (identity, changing professionalism); Learning (re-negotiating roles with technology)
Sociomateriality, technology–work coupling	(17, 18, 23, 48–50)	Information systems, organizational technologies	Organizations (incl. healthcare)	Professionals, managers, teams	Execution (routines, flexible practices); Governance (structures and rules constituted in use); Learning (ongoing adaptation, imbrication); Experience (human–technology agency)
Digital competence, education, acceptance	(7, 8, 51–54)	Digital health, e-learning, IT in general	Health professions education, clinical training, organizational IT	Students, health professionals, IT users	Learning (competence frameworks, training, evolution of skills and attitudes, cognitive load); Experience (usefulness, ease of use, acceptance); Execution (effective uptake in practice)
Job crafting, paradox, future of work	(14, 55–57)	General work and organizing (applied to digital transformation)	Organizations, including healthcare	Workers, managers, occupational groups	Experience (tensions, job crafting, human side of change); Governance (managing paradoxes, boundary work); Learning (how people shape and make sense of changing work)

### 3.1 Work execution

Work execution refers to how tasks are distributed, sequenced, and temporally organized. Digital technologies rarely eliminate work. More often, they reorganize it by introducing new tasks, redistributing coordination labor, and reshaping temporal sequencing of activities. Research shows that digital systems such as EHRs expand documentation and coordination activities alongside clinical tasks, without reducing overall workload (20, 21, 27, 28). These expansions frequently extend beyond the clinical encounter, stretching responsibility across time and settings (29). The analytical value lies in distinguishing changes in both the *content* and *organization of work*. Information-mediated execution makes data production a prerequisite for work to count as legitimate activity (36).

### 3.2 Work experience

Work experience captures how digitalized work is lived, interpreted, and evaluated by workers. This includes autonomy, wellbeing, cognitive load (54) and dignity. Dignity is understood as an outcome of how work is organized, valued, and recognized within organizational arrangements (45). Digital technologies shape experience not simply by adding workload, but by altering what it means to do one's job well, how responsibility is attributed, and how expertise is recognized (22, 25). When professional action must be rendered defensible through data and metrics, dignity may be reinforced or undermined depending on whether expertise, and judgment are recognized or reduced to informational proxies (37).

### 3.3 Work governance

Work governance refers to how work is standardized, monitored, evaluated, and held accountable. Digital technologies embed governance directly into work processes through metrics, audit trails, and performance indicators (19, 26). Governance is no longer external to work but enacted through routine information-mediated practices. Professional judgment is continuously assessed through data traces and algorithmic proxies (47). Recent syntheses highlight that these mechanisms are not applied uniformly. Surveillance and metric-based evaluation intensify as we move down occupational hierarchies, with frontline workers facing tighter constraints, while professional status offers greater protection for physicians (43). Analytically separating governance reveals how digital systems simultaneously rely on professional judgment and subject it to continuous monitoring and comparison.

### 3.4 Work learning and adaptation

Work learning and adaptation, captures how workers and organizations adjust, appropriate, and reshape digital technologies and work practices through use. This includes experimentation, workarounds, informal innovation, job crafting, skill development, and feedback into redesign (30, 49, 55, 58, 59). This dimension helps explain why digital systems rarely stabilize and why their effects evolve over time. Reviews of digital health competencies suggest that workforce learning extends beyond basic digital literacy to include information management, data governance, and AI-supported judgment (51, 52). Treating learning and adaptation as analytically distinct allows the framework to capture productive forms of agency that are not reducible to experience, resistance, or compliance. It also provides a basis for analyzing how human–technology collaboration is refined, recalibrated, and contested (42).

### 3.5 Positioning against established frameworks

The framework complements rather than replaces established approaches. Work system models like SEIPS (32, 33) provide taxonomic structure for patient safety analysis. The present framework shares its sociotechnical orientation but focuses on dynamic relationships among dimensions. Whereas SEIPS asks what elements comprise the work system, the four-dimensional lens asks how changes spread across dimensions. Implementation complexity frameworks such as NASSS (60) help explain why technologies struggle to achieve adoption. The present framework instead examines how work is reconstituted regardless of implementation success and addresses questions that persist after adoption. Health information technology safety frameworks such as SAFER (31) prescribe safe practices and offer essential operational guidance for reducing technology-related risks. This framework focuses on why safety issues emerge from the structural dynamics of digitalized work and on why safe practice is difficult to sustain over time.

### 3.6 Analytical leverage and operationalization

The framework's analytical leverage operates at three levels. First, it enables diagnosis of structural misalignment as the source of persistent tensions and reframes burnout, resistance, and instability as predictable outcomes rather than individual failures or technological shortcomings. Second, it makes visible trade-offs and unintended consequences as inherent to how dimensions are coupled, which calls for ongoing negotiation rather than elimination. Third, by treating learning as constitutive rather than transitional, it explains why digital systems remain contested and continuously reconfigured over time.

The framework also generates concrete diagnostic questions. Have efficiency-oriented tools increased total work time, and where has labor been displaced? Do workers experience expanded information access as enabling or as exposing? Are workarounds treated as deviance or as signals of improvement? How does documented practice diverge from observed practice? These questions can be translated into testable propositions, including expectations that efficiency tools increase cognitive load even when they reduce task time, that experiences of surveillance vary by occupational status, that tighter governance is associated with growth in hidden workarounds, and that systems with low tolerance for variation generate more shadow practices.

Importantly, tensions do not affect all workers equally. Digital transformation redistributes tasks, recognition, surveillance, and learning opportunities in ways that tend to reinforce existing occupational hierarchies (43, 44). Execution burdens concentrate among lower-status workers as documentation tasks shift downward. Experiential costs such as burnout receive attention primarily when they affect physicians, while comparable harms to nursing and administrative staff remain less visible in research and policy. Governance mechanisms impose more intensive algorithmic monitoring on frontline and clerical workers, while professional status continues to shield physicians from the most constraining forms of digital control. Learning opportunities also remain stratified by occupational position. Information-mediated work thus becomes a site where existing inequities may be reproduced or amplified through technological infrastructure, which requires deliberate attention to distributive effects in both research and implementation.

The tensions generated through information-mediated coupling are not problems to be solved but paradoxes to be navigated (56). The framework identifies four characteristic paradoxes. The efficiency–intensification paradox captures how technologies introduced to improve efficiency simultaneously intensify cognitive and temporal demands. The empowerment–surveillance paradox reflects how systems that expand information access for workers also enable monitoring. The innovation–compliance paradox describes how governance mechanisms that secure standardization constrain the variation needed for innovation. The adaptation–deviation paradox recognizes that local adjustments that improve practice may also constitute deviations from formal protocols. These paradoxes are structural features of digitalized work, since the same informational practices that generate benefits in one dimension produce costs in another. Identifying specific configurations supports diagnosis and suggests that sustainable improvement depends on managing trade-offs rather than attempting to eliminate them.

Recognizing these paradoxes does not imply that digital transformation inevitably undermines work. Emerging evidence points to conditions under which digitalization can enhance autonomy, safety, and dignity. Participatory design processes that involve frontline workers in system configuration can align execution demands with experiential realities (30). Protected time for documentation rather than expecting it to occur alongside or after clinical work, directly addresses efficiency–intensification dynamics. Transparent governance arrangements that allow workers to see and contest the metrics applied to them can mitigate surveillance effects and preserve professional discretion. Learning-oriented cultures that treat workarounds as signals of innovation rather than compliance failures create space for productive adaptation. The framework therefore offers not only a diagnostic tool for identifying tensions but also design guidance. Sustainable digital transformation requires explicit attention to cross-dimensional alignment and particular vigilance for how benefits and burdens are distributed across occupational hierarchies. The central issue is not whether to digitalize, but how to configure digital work systems in ways that preserve the conditions for dignified, expert practice.

## 4 Analytical fragmentation in existing research

Research on digitalization and work has produced valuable insights, but these contributions remain scattered across domains and traditions. Most studies concentrate on specific dimensions without examining how changes in one affect the others (38). This fragmentation has important consequences. Persistent tensions, such as worker resistance and burnout, are often attributed to poor design or implementation failures. The framework advanced here suggests a different diagnosis. These tensions reflect limitations in how the problem itself has been conceptualized.

A substantial body of research examines how digital technologies reorganize tasks through concepts like routines and affordances (18, 20, 21). These accounts, however, often marginalize human consequences. When documentation and data production become primary evidence of performance, they fundamentally alter how professionals exercise judgment, experience recognition, and account for their work. A separate literature centers on professional experience, including autonomy, identity, and dignity, showing how digital systems reshape the moral underpinnings of work (25, 45, 46). By treating task configurations as given, this work overlooks how experiences are shaped by what workers are required to do and how their work is evaluated. A third body of research focuses on governance, demonstrating how metrics and audit systems embed accountability into everyday practice (19, 26, 57). Governance-centered accounts, however, risk portraying workers primarily as objects of control while underplaying their capacity to learn and adapt.

Each perspective therefore offers a partial explanation. Digital transformation promises efficiency while producing intensification, enables standardization while constraining judgment, and creates visibility while rendering aspects of work invisible. These outcomes are predictable when dimensions operate in misalignment (9, 17, 61). The framework reorients analysis toward understanding how work dimensions stabilize, destabilize, and evolve as professionals navigate information-mediated work.

## 5 Implications

The framework advances theorizing by analytically separating dimensions that are often collapsed in existing accounts. It moves beyond explanations that attribute outcomes solely to technological features or professional attitudes and highlights how consequences emerge from misalignments among work dimensions. It contributes to debates on digital control by specifying information-mediated work as the coupling mechanism, shifting attention from technology as an external driver toward everyday practices that render work visible, accountable, and improvable. By conceptualizing learning as structural rather than transitional, it enables theorization of digital transformation as ongoing co-evolution.

Empirically, the framework suggests that studies focused on single dimensions risk overlooking the dynamics that generate enduring tensions. Research should attend to simultaneous changes across dimensions and to how configurations evolve over time. This calls for longitudinal approaches that capture information-mediated practices as sites of intersection, moments of misalignment and adaptation, and temporal processes through which technologies and work arrangements are reshaped.

## 6 Discussion and conclusion

A crucial insight follows. Many tensions in digitalized healthcare arise not from how technologies are designed but from misalignments among work dimensions (22). Consider a common scenario, in which clinicians spend increasing time on documentation, while managers prioritize metrics, yet neither work design nor governance arrangements recognize clinical judgment or allows time for learning and adaptation. Worker resistance, burnout, and stalled implementation are not individual failings or technological shortcomings. They are symptoms of structural imbalance. Without understanding how dimensions misalign, we misdiagnose problems and pursue solutions that address symptoms rather than causes.

By treating learning as a core structural element, the framework challenges the assumption that digital transformation reaches some stable endpoint once systems are deployed. In healthcare, where work is interdependent, morally charged, and time-pressured, digital systems remain subject to reinterpretation and modification. Recognizing learning as constitutive helps explain why digital infrastructures continue to evolve and why their effects remain contested over time.

How work dimensions align or misalign shapes what professionals experience as recognition, accountability, and worth, which together constitute dignity at work. When work becomes visible primarily through data and metrics, clinicians remain essential, but their judgment is constrained by what can be measured. Documentation designed for accountability can inadvertently signal that unmeasured aspects of care do not count. Placing dignity at the center shows that digital transformation stakes extend beyond efficiency to workers' sense of themselves as valued contributors with moral standing.

The urgency of this perspective has intensified in the post-pandemic period. Digital systems expanded rapidly, often under pressure and without sufficient attention to how work was reorganized. Many organizations are now confronting the consequences, such as clinician burnout, rising turnover, quality concerns, systems that are simultaneously indispensable and dysfunctional. If we frame these as implementation failures, we miss the structural dynamics that sustain them. A work-centered perspective instead invites different questions. Not “How do we get clinicians to adopt this system?” but “How are we reconfiguring work dimensions, and are those reconfigurations sustainable and dignifying?”

The framework offers no quick fixes, but it provides diagnostic orientation. When digital transformation generates persistent tension, the problem rarely lies in a single dimension. It lies in misalignments among them. Addressing these conditions requires simultaneous attention to how execution, experience, governance, and learning are configured and how those configurations evolve. For healthcare leaders, policymakers, and implementation teams, this means moving beyond technology procurement and training models toward sustained inquiry into whether digital systems enable or erode the conditions under which dignified, expert work can flourish.

Information-mediated work, including documentation, coding, classification, and data verification, functions as the coupling mechanism linking work execution, work experience, work governance, and work learning and adaptation. This coupling has three defining properties. It is constitutive, in that information practices define what counts as legitimate work; transductive, in that changes in one dimension propagate to others through informational practices; and asymmetric, in that datafied work becomes systematically more visible than non-datafied work. Bidirectional arrows indicate mutual influence among dimensions, while tensions represent paradoxes to be navigated rather than problems to be resolved.
